# Service Life of Pre-Tensioned Concrete Structures in a Chloride Environment on the Example of an Aluminium Foundry Building

**DOI:** 10.3390/ma17122985

**Published:** 2024-06-18

**Authors:** Jarosław Michałek, Filip Gago

**Affiliations:** 1 Faculty of Civil Engineering, Wroclaw University of Science and Technology, 50-370 Wrocław, Poland; 2 Faculty of Civil Engineering, University of Žilina, 010 26 Žilina, Slovakia; filip.gago@uniza.sk

**Keywords:** chloride environment, chloride concentration measurements, prestressed concrete construction, durability

## Abstract

This paper describes a study of the chloride content in the concrete lagging of prestressed concrete elements of the roof structure of an aluminium foundry building. Sources of chloride pollution in industrial facilities are discussed. Methods for collecting dust deposited on the structure and sampling concrete for chloride concentration testing are presented in detail. The test methods used and the apparatus used to assess the chloride content at the thickness of concrete reinforcement lagging are presented. Investigations of the chloride content in the concrete of the reinforcement cover showed a very high concentration of chloride in the near-surface layer, depending on the location of the element in relation to the source of chloride emission into the environment. In contrast, the concrete of the deeper layers of the lagging contained very small amounts of Cl^−^ chlorides. The results of the chloride content of the lagging concrete were plotted against the distance from the surface of the specimen and approximated by the function *C*(*x*,*t*) based on Fick’s second law and its solution. A satisfactory fit of the course of this function to the experimental results was obtained. Based on the obtained *C*(*x*,*t*) function, the durability of the main structural components of an aluminium foundry industrial hall operating in a chloride environment was estimated.

## 1. Introduction

The commonly held view that concrete is a material that does not need repairs and is infinitely durable has changed in recent years. Even though the durability of concrete, reinforced concrete and prestressed concrete structures is high, it is not infinite, and after some service life, concrete structures show all kinds of damage and chemical contamination and need to be repaired or renovated (e.g., [1,2,3,4,5,6]). The causes of the damage can be of chemical nature (the aggressive action of acids, sulphates, chlorides, soft waters, carbon dioxide, ammonium, magnesium salts, and petroleum derivatives) or non-chemical nature (excessive deformations, cracks, fractures, cavities, and concrete scaling caused by exceptional static or dynamic loads or local overloads).

A concrete structure serving in chemical action conditions can be slightly (superficially) contaminated, partially contaminated (to a certain depth of the reinforcement cover), or completely contaminated (down to the reinforcement zone or deeper). If harmful substances penetrate into concrete, one should assume the so-called chemical leakage time t_ch_ [7,8], i.e., the time after which the aggressive agents penetrate so deep into a concrete element that its proper behaviour is no longer possible (in the case of substances harmful to reinforcement steel, t_ch_ is assumed to be the time in which the contamination front reaches the reinforcement) [9]. If time t_ch_ is longer than predicted service life t_ex_ of a structure, the structure is safe, whereas when it is shorter, in order to extend it, one should apply surface treatment to the concrete or limit the access of the aggressive environment.

In industrial facilities, concrete structures are often exposed to contamination with chemical compounds present in their service environment, e.g., [10,11]. It is in the interest of the user of an industrial facility to take care that the reinforced or prestressed concrete structure is not exposed to excessive quantities of aggressive substances, particularly those aggressive towards reinforcement steel. Then the concrete cover will effectively protect conventional and prestressed reinforcement against corrosion in the course of the design and service life of the structure.

Chlorides and chlorine compounds present in the service environment of a concrete structure are a highly dangerous corrosion factor since, in favourable conditions, they quickly penetrate into the concrete. In concrete, the penetration of chlorides coming from the environment forms a chloride content profile characterised by a high content close to the outer surface, which decreases with depth. The actual profile at a particular point of a given structural member depends on many factors. The main factors are linked to the properties of the concrete, the chloride solution transport mechanisms, the moisture content of the concrete, and the chloride concentration in the environment.

The transport of chlorides through reinforced concrete covers can take place via the mechanisms of diffusion, capillary rise, penetration (when under pressure a solution penetrates into saturated concrete), and migration (the transport of charged ions present in the pore solution under the influence of an electric field) [12,13,14,15,16,17]. Diffusion occurs due to the presence of a concentration gradient—when the surface of concrete saturated with water comes into contact with a solution containing chlorides. Chlorides penetrate through the filled with water pores of the cement paste. The diffusion of chlorides in saturated pores also takes place during drying [16,17,18,19]. Capillary rise consists of the penetration of a liquid into empty or partially saturated pores of a hydrophilic material as a result of a negative pressure in the pores. When the surface of unsaturated concrete comes into contact with a solution containing chlorides, this solution (and also the chloride ions dissolved in it) is quickly absorbed by the concrete. When the drying period is extended, this seems to increase the penetration of chlorides not through continuous diffusion during the drying phase but through capillary sorption in subsequent cycles of wetting [18]. Basically, all the transport parameters depend on the microstructure of the concrete.

In existing structures, the transport of chlorides through concrete often takes place through a combination of transport mechanisms [12]; for example, when a structural element is subjected to wetting-drying cycles [16]. During wetting, it is subjected to chloride solution capillary absorption (then possibly diffusion in a wet period), while in dry periods, water evaporation results in the accumulation of chlorides close to the surface.

Thus, the durability of concrete depends on the long corrosion initiation stage [20], which is a function of the way in which chlorides are transported, the environmentally determined concentration of chlorides on the surface of the concrete, the thickness of the reinforcement cover, the tightness of the concrete, and the critical free chloride content in the concrete [7,21,22,23]. At the moment when the chloride concentration reaches the critical value C_crit_, the corrosion of the steel begins [21,24,25,26,27].

The review of the literature on the subject shows that research on the chloride corrosion of steel in concrete focuses more on reinforced concrete structures than on prestressed concrete structures. This is very curious, especially as the rate of corrosion increases with stress in prestressing steels, which makes prestressing concrete structures of particular interest. Furthermore, there are few studies conducted on existing structures.

This paper presents the results of research on the degree of contamination with chlorides of prestressed concrete girders and precast ribbed floor elements (TT slabs), based on a case study of the structure of an existing operational industrial shed used for the production of aluminium castings for the automotive industry. The sources of the emission of chlorides and chlorine compounds in various branches of industry, with a special focus on casting and metallurgical facilities, are discussed, and the effect of the action of chlorides on reinforced concrete structures and prestressed concrete structures is described. The method of taking concrete cover samples for testing and relevant physical and chemical tests are described. In particular, experimental tests of the chloride ion content in concrete cover layers and the assessment of the chloride concentration relative to the critical values are discussed. The method of selecting theoretical curves (according to Fick’s law) for the approximation of experimental distributions of chloride concentrations across the thickness of the cover is described, and the service lives of the structural components of the industrial shed are determined.

## 2. Chloride Emission Sources in Industrial Facilities

Chlorine is used as a cheap chlorinating and oxidising agent in the production of, i.a., plastics, synthetic rubber, organic solvents, and chlorine for the bleaching of flax, cotton, and paper pulp [28], water and sewage decontamination [29], and metal regeneration. A chlorine and chloride compound emission hazard can occur in, i.a., plants producing explosives, fireworks, and matches (because of the use of potassium chlorate KClO_3_ and sodium chlorate NaClO_3_), in plants manufacturing piston or rotary refrigerating-compressing equipment, and in small household refrigerators (refrigerants according to the American Society of Heating, Refrigerating, and Air-Conditioning Engineers: methylene chloride CH_2_Cl_2_—R30, methyl chloride CH_3_Cl—R40, ethyl chloride C_2_H_5_Cl—R160 [30]).

Potassium chloride KCl is used in potassium fertiliser production, in the manufacture of soap, potassium glass, and welding agents manufacture, and in medicine and the pharmaceutical industry [31], while mercury chloride HgCl_2_ is used in the production of seed dressings, insecticides, and disinfectants, in steel etching and wood preservation, in the photographic industry, and in lithography and engraving [32]. Iron chloride FeCl_2_ is used in various metallurgical processes, while iron trichloride FeCl_3_ is used in metallurgy to process copper and silver ores [33,34].

In the casting industry, admixtures and impurities are removed from aluminium alloys using fluxing, floatation, and filtration, which are conducted in a pre-treatment crucible and/or a foundry furnace, a degasser, and a filtrating unit, respectively [35]. Chlorine is the only gas that is used as a fluxing agent to remove alkaline impurities. Reacting directly with the metal, it forms alkaline chloride. In alloys containing magnesium, it reacts with the latter, forming magnesium chloride, which then reacts with the alkaline metal, forming alkaline chloride. In both of these cases, alkaline chloride separates from the alloy, floating on its surface. Chlorine is added to aluminium as a pure gas or mixed with an inert gas, such as argon or nitrogen. After it leaves the foundry furnace, the molten aluminium is transferred to a degasser. The purpose of degassing, which is conducted in a closed tank, is to lower the overall hydrogen level in the liquid aluminium through floatation. The presence of chlorine in the supplied gas is essential since, without it, the hydrogen level cannot be brought down. Moreover, chlorine initiates the further removal of alkaline metals. Currently, there are no substitutes for chlorine in degassing practices [35]. The filtration of minute particles of impurities in aluminium after degassing is the final stage in aluminium processing before the casting of end products.

This means that in industrial facilities in which chlorine is used in manufacturing processes, the air and the structural elements are contaminated with chlorine due to the emission of chlorine and hydrogen chloride gases (among others: [36,37,38,39,40]).

## 3. Damaging Action of Chlorides

The steel in concrete is protected by the alkaline solution contained in cement paste pores, which fosters passivation, i.e., the formation of a self-contained thin protective layer of oxide on the surface of the steel [12,41]. In these conditions, the corrosion rate is negligible, even when the concrete is saturated with oxygen and moisture. However, corrosion can take place when the passive layer is removed or locally damaged. The damaging action of chemical compounds towards reinforced concrete structures and prestressed concrete structures consists in neutralising the concrete cover’s protective properties towards the steel, whereby the latter does not produce a passivation layer [20,27,42]. The main factors causing the neutralisation of the concrete’s protective properties towards the steel are acid gases, including sulphur dioxide, nitrogen oxides, and chlorine, and, at the access of moisture, hydrogen sulphide and hydrogen chloride, and compounds containing electrolytes, mainly sulphates and chlorides (CaCl_2_, MgCl_2_, NaCl).

The damaging action of chlorine on concrete consists in its reaction with the lime Ca(OH)_2_ contained in it. As a result, readily soluble calcium chloride and water are formed [27,28]. The concrete then becomes porous and permeable. The chlorides acting on the concrete can form a harmless complex compound, i.e., Friedel’s salt (stable only in an alkaline environment), with the tricalcium aluminate C_3_A and water present in the concrete. When the pH reaction in the concrete is lowered, some quantity of the chlorides can transform into a form aggressive towards the steel.

The passivation of the reinforcement steel in a basic (alkaline) environment with a pH > 9 consists in the formation of a thin layer of iron(II) hydroxide on its surface, which protects the steel against corrosion. As a section of the passive layer of the reinforcing steel is damaged, reactions begin between the anodic (damaged, stripped of the passive layer) and cathodic areas (still containing the passive layer) connected by an electrolyte in the form of pore water in the hardened cement slurry. According to [25,26,42,43,44,45], the positively charged iron ions Fe^2+^ on the anode pass into the solution, while the negatively charged electrons e- pass through the steel to the cathode, where they are absorbed by the electrolyte constituents and combine with water and oxygen, forming hydroxide ions OH^−^:

Fe → Fe^2+^ + 2e^−^

Fe^2+^ + 2OH^−^ → Fe(OH)_2_ (iron(II) hydroxide)

The latter ions pass through the electrolyte and combine with iron ions, forming iron(II)hydroxide, which, as a result of further oxidation transforms into rust:

4Fe(OH)_2_ + 2H_2_O + O_2_ → 4Fe(OH)_3_ (iron(III)hydroxide)

The reaction 4e^−^ + O_2_ + 2H_2_O → 4OH^−^ takes place on the cathode, which means that oxygen is consumed and water (essential in order for the process to continue) is regenerated. Consequently, there is no corrosion in dry concrete [25]. In order for the above equations to be feasible, the pH of the water must be below 11.5. The total or partial damage to the passivation layer (the change of iron(II)hydroxide to iron(III) hydroxide) causes the so-called activation of the steel, which may rust despite the thick layer of concrete cover.

The possibility of corrosion is inhibited by the highly alkaline properties of concrete. Owing to this, besides rebars, layers rich in calcium form in the interfacial zone separating the concrete from the reinforcement. The main role of the calcium-rich layers is to ensure an OH reservoir around and in the vicinity of the reinforcement steel. This contributes to the suppression of the possibility of steel corrosion owing to the maintenance of a high pH in the pore water of the concrete around the reinforcement steel (anodic passivation). However, when the OH level is not sufficiently high or when chlorides are present in a sufficiently large quantity, the chlorides can attack local residua of Fe(OH)_2_, leading to local chemical reactions.

In order for the corrosion of the steel to begin, the concrete’s passive layer must be neutralised. Chloride ions activate the surface of the steel, creating an anode (the corroding steel), and the surface being passivated becomes a cathode. The reactions occurring in this process are as follows [25]:

Fe^2+^ + 2Cl^−^ → FeC1_2_

FeC1_2_ + 2H_2_0 → Fe(OH)_2_ + 2HCl

In this way, Cl^−^ is regenerated so that the rust does not contain chlorides, although iron chloride is formed in the intermediate phase. The electrochemical cell requires a connection between the anode and the cathode via pore water, as well as via the reinforcement steel itself. The system of pores in the hardened cement paste is the main factor influencing corrosion. The total increase in the rate of corrosion of the steel depends on the ratio of anodic/cathodic sites and the electrical resistance of the concrete [46]. The electrical resistance of the concrete to a high degree depends on its moisture content, the ionic composition of the pore water, and the continuity of the pore system in the hardened cement paste.

There are two consequences of corrosion. Firstly, the corrosion products occupy a volume several times larger than that of the original steel, whereby their formation generates tensile stresses in the concrete cover, which may lead to cracking and chipping in localised areas or to delamination [47,48]. This facilitates the penetration of aggressive agents into the steel, which ultimately leads to an increase in the rate of corrosion. Also, the adhesion of the reinforcement to the concrete may decrease (this particularly applies to pre-tensioned concrete structures). Secondly, the advancement of corrosion on the anode reduces the cross-sectional area of the steel and so reduces the load-bearing capacity of the affected structural member. After corrosion is initiated, a very aggressive environment is created within local corrosion areas (corrosion pits), while the protective layer on the surrounding passive surface is maintained (and even strengthened). Corrosion within the corrosion pits can reach very high penetration coefficients (up to 1 mm per annum) in wet and strongly chloride-contaminated structures [12], and the non-allowable reduction in the reinforcement cross section can be reached in a relatively short time.

The high-strength steels used in prestressed concrete are rather susceptible to corrosion. Because of the high stress in the prestressing tendons, even a slight corrosion attack can result in their damage [49,50]. A structural failure caused by stress corrosion is initiated by the cracking of the prestressed steel, while pitting corrosion reduces the cross-sectional area of the prestressing steel and, consequently, the structure’s load-bearing capacity.

## 4. Structure Description and Environmental Factors

**Figure 1:**
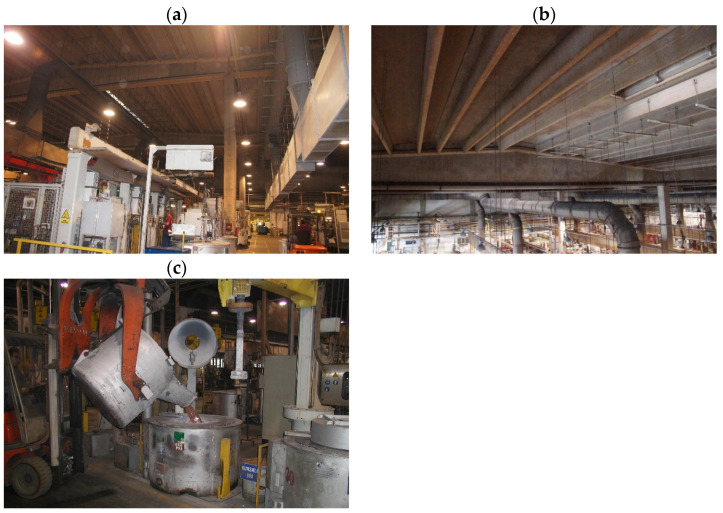
Aluminium foundry shed: (**a**) general view of shed’s interior, (**b**) shed’s roofing components—pre-tensioned concrete slabs TT-62 resting on double-tapered beams, (**c**) station for purifying liquid aluminium with gaseous chlorine.

The aluminium foundry building is a 75.0 m long, three-bay (3 × 22.5 = 67.5 m) post-and-beam prefabricated industrial shed [51]. Pre-tensioned slabs TT-62 with a span of 14.75 m, resting on pre-tensioned double-tapered beams with a span of 22.5 m constitute the roofing of the shed (Figure 1). The shed’s roof is supported by 10.0 m high reinforced concrete columns 0.6 × 0.6 m in cross section, set in pocket footings. The shed’s enclosure is made up of 14 cm thick uninsulated prefabricated reinforced concrete panels resting on corbels.

A dry chlorine environment classified as XD1 (surfaces of concrete with a moderate moisture content, exposed to the action of air chlorides [52,53]) occurs inside the shed. The occurrence of environment XD1 in the whole shed, and so also at the contact between the shed’s internal atmosphere and the enclosure (the roof, the curtain walls, the windows, the doors, the gates, the roof ventilators, and the ventilators in the walls), is contingent on the proper thermal performance of the building fabric, protecting the latter against air water vapour condensation when a dew point arises. If periodically water condenses on building fabric elements (e.g., on the uninsulated exterior walls or due to periodical repair stoppages), these elements serve in the XD3 environment (cyclic wet and dry conditions [52,53]).

Among the environmental factors that occur in the aluminium foundry shed, one should mention:

- the acid gases (Cl_2_, CO_2_, HCl, HF, F_2_, SO_2_, NO_2_) and dusts (mainly chlorides and metal fluorides) released in the manufacturing process,
- the elevated temperature (usually above 20 °C),
- the low air humidity (below 50%).

On the basis of the above conditions, the exposure class can be assumed, according to [52,53], as XD1. F, or reinforced concrete, and prestressed concrete. The aggressive agents are the acid gases present in the air. They can be divided into three groups, depending on the kind of products they form as a result of the reaction with the components of concrete [54]. The particular groups comprise gases, causing the formation of:

1. practically insoluble or poorly soluble salts—as a result of the reaction of atmospheric CO_2_ with calcium hydroxide (a constituent of concrete), poorly soluble CaCO_3_ forms and the concrete undergoes partial neutralisation, whereby the pH of the concrete cover can decrease to a value at which steel depassivation takes place (a pH below 11);
2. poorly soluble calcium salts, which, as they crystallise, bond considerable amounts of water, which may lead to an increase in their volume to a degree resulting in damage to the reinforcement concrete cover (SO_2_, H_2_S),
3. highly water-soluble salts that can penetrate into concrete beyond the neutralised zone; this poses a particularly serious hazard when they are depassivators, destroying passive films on the surface of the reinforcement (Cl_2_, HCl).

The gases belonging to the first group and the second group cause reinforcement corrosion when the neutralised layer reaches the surface of the reinforcement. In the case of the gases belonging to the third group, reinforcement corrosion may begin before the neutralisation zone reaches the reinforcement cover. If the concentration of the third group of salts reaches critical values at the surface of the steel, the destruction of the protective films on its surface will begin.

Unlike the diffusion of chlorides, the diffusion of CO_2_ can take place in nearly dry concrete, while in highly damp concrete it almost disappears. The diffusion of chlorides depositing on the structure’s surface can take place in capillary voids filled with water. In concrete that has been dried, this process is arrested. As the moisture content increases, the diffusion of chlorides intensifies, and it will proceed at the fastest rate when the concrete is fully saturated with water.

According to codes [52,53], the structure of the aluminium foundry shed meets the requirements of not only environment exposure class XD1, but also XD2 and class XD3. The requirements of codes [52,53] concerning minimum cover thickness (c_min_ = 50 mm for prestressed structures at the service life of 50 years) and crack width (w_lim_ ≤ 0.2 mm) are the same for all three XD classes, and the concrete meets the requirement: w/c < 0.45 and contains at least 320 kg of cement in 1 m^3^ of concrete.

## 5. Results of Chemical Tests on Dust Accumulated on Structure

### 5.1. Sampling for Testing

Samples for testing were taken from various places in the shed and from its various structural members where large amounts of dust had accumulated. The samples were stored in tightly closed vessels in order to protect them against changes in their chemical composition and moisture content. Table 1 shows the sampling sites on the roof girders, the kind of substrate, and macroscopic and microscopic descriptions of the taken samples. The tested loose dusts were a mixture of aluminium alloy reaction products and air pollutants inside the shed, mainly process dusts.

### 5.2. Research Methodology

The loss on drying at the temperature of 105 °C and the loss on ignition at the temperature of 1000 °C after the samples had been dried at 105 °C were determined (Table 2). Five-percent solutions of the tested samples (with their original moisture content) in distilled water and in a hydrochloric acid solution (HCl:H_2_O = 1:2) were prepared. The solutions were heated up to boiling and then kept for 2 h at a temperature ensuring gentle boiling. After 24 h the suspensions were filtered through filters of medium hardness. After washing the residue on the filters and drying it at 105 °C, the percentage of insoluble matter in the distilled water and in the hydrochloric acid solution was determined (Table 2). The obtained water extracts were subjected to further tests to determine the pH of the water filtrates (Table 2) and the Cl^−^, SO_4_^2−^, Ca^2+^, Mg^2+^, Fe^3+^, and Al^3+^ ions content in the water filtrates according (Table 3) to standard [55].

Sample no. 1 taken over the station for purifying liquid aluminium by blowing the latter with gaseous chlorine (the place with the highest emission of harmful substances), was used in instrumental chemical tests. A spectrochemical analysis and an X-ray phase analysis were carried out for sample no. 1 with its original moisture content. The tests showed that the Cl^−^ ions content and the SO_4_^2−^ ions content in the sample amounted to respectively 20.4% and 2.14%. The other chemical elements and ions (Mg, F^−^, Nor, N(NH_4_^+^) which may destructively affect concrete were found to be present in the amount of 0.065 ÷ 0.624%. A semi-quantitative chemical analysis of the other elements showed that the largest fractions in sample no. 1 were those of silicon, calcium, aluminium, iron, potassium, and sodium compounds.

**Figure 2:**
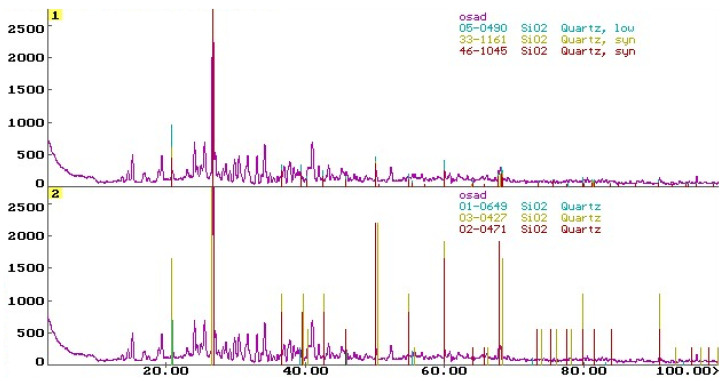
X-ray phase analysis of sample no. 1—comparison of the diffraction pattern of the sample with the standard diffraction pattern for SiO_2_; all significant peaks originate from SiO_2_.

A diffraction phase analysis (Figure 2) showed mainly SiO_2_ and small quantities of aluminium sulphate, hydrate calcium and aluminium chlorides, and potassium and magnesium chlorides to be present in the crystalline form in sample no. 1. The other compounds occurred in amorphous and subcrystalline form or in an amount that made identification impossible.

A Philips V’PERT X-ray diffractometer equipped with a PW1830 generator, PW3710 control module, and PW3719 counter was used for the tests. The radiation used was CuKα (λ = 0.15418 nm), a vertical goniometer, and a counter recording of the angle and position of the reflector. The measuring range of the angle 2θ was 3 ÷ 100^0^ (horizontal axis on the graph—Figure 2), the step (counter travel rate): 0.050, single pulse count time: 2 s, voltage: 40 kV, current: 30 mA. The vertical axis shows the intensity of the substance (Figure 2). The processing of the experimental data, i.e., background removal, calculation of position, integration intensity, and peak heights, was carried out using the DHN-Power Diffraction System computer programme. Identification of the individual crystalline phases of the substances present in the samples was carried out by comparing the experimental diffractogram of the test sample with the reference diffractogram found in the ICDD PDF-2 database [56].

Those of the samples that are soluble in water constitute a factor that is aggressive towards the reinforced concrete. The 5% filtrates of the water solutions of samples 1 and 2 taken from the concrete substrate show a pH of 5.10 ÷ 5.38 (Table 2). The water solubility of the (not dried) samples is in the range of 37.2 ÷ 64.3% wt., and it is decidedly the highest for sample 1 (Table 2). The percentage of Cl^−^ ions passing into the water solutions from the samples with their original moisture content is in the range of 11.50 ÷ 19.66%, and that of SO_4_^2−^ ions is in the range of 2.64 ÷ 4.31% (Table 3).

Spectrochemical tests carried out for sample no. 1 corroborated the results of the analyses of the water extracts of the samples, particularly as regards the chloride ion content—the main danger to the durability of reinforced concrete (the spectrochemical analysis showed the Cl^−^ content of 20.4% in the whole sample, while in the water extracts this content amounted to 19.66%, which means that nearly all the chlorine had passed into the solution).

### 5.3. Discussion of Dust Test Results

On the basis of the chemical tests of the composition and aggressiveness of the compounds and deposits taken from various structural members of the aluminium foundry shed, one can state that the tested dusts at a relative air humidity below 50% (when the foundry’s full production capacity is used, the temperature under the shed’s roof amounts to +25 ÷ 30 °C and the relative air humidity does not exceed 25%) do not pose a significant hazard to impermeable concrete. The diffusion of the chlorides settling on the structure’s surfaces can take place in capillary voids filled with water. Investigations [54] have shown that at a relative air humidity below 75%, the continuity of the water films in capillary voids is broken and the diffusion of chlorides in the concrete sharply decreases.

The dust accumulated on structural members can pose a significant hazard to a reinforced concrete structure or a prestressed concrete structure only when they become damp to a degree that makes the penetration of Cl^−^ ions into the concrete possible. This means that particularly the structural elements near which the relative air humidity exceeds 75%, i.e., in the vicinity of skylights, gates, and uninsulated exterior walls, are in danger. In winter, water vapour condenses (dew point zones) on these elements. Chlorides dissolve in this water and penetrate into the (particularly cracked) concrete, damaging its structure. Also, an adverse effect of repair stoppages (when production was stopped altogether, the furnaces were shut down, and the liquid aluminium casting ladles were cooled down) was observed. After a few days of airing the shed to quickly bring down the temperature of the process facilities, marks left by falling drops of dark brown liquid appeared on the shed’s floor along the ribs of the roof slabs and girders. Tests showed that the dark brown gunk collected from the surface of the roof’s structural members (Figure 2) contained about 13% of Cl^−^ ions.

## 6. Testing of Reinforcement Concrete Cover

### 6.1. Taking Samples

**Figure 3:**
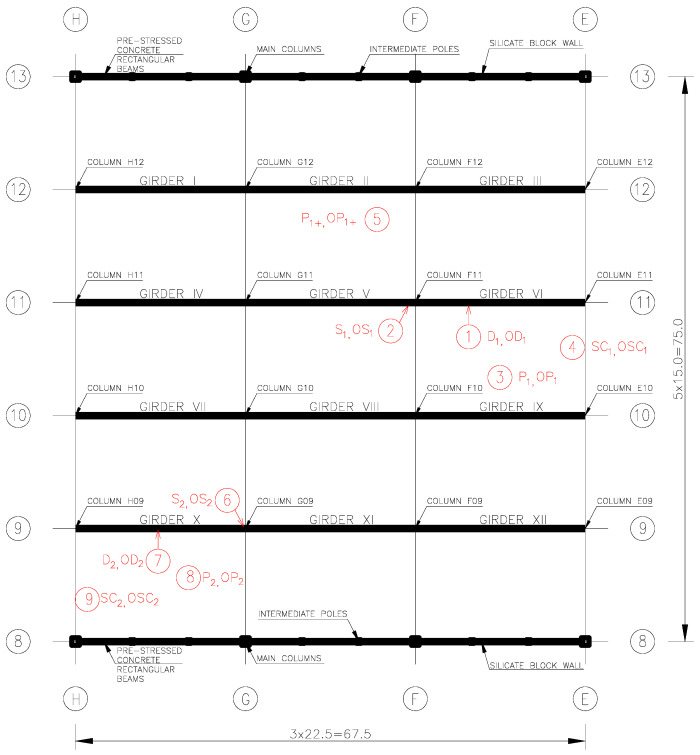
Plan sketch of the aluminium foundry hall construction with marked sampling locations for testing (description in text).

**Figure 4:**
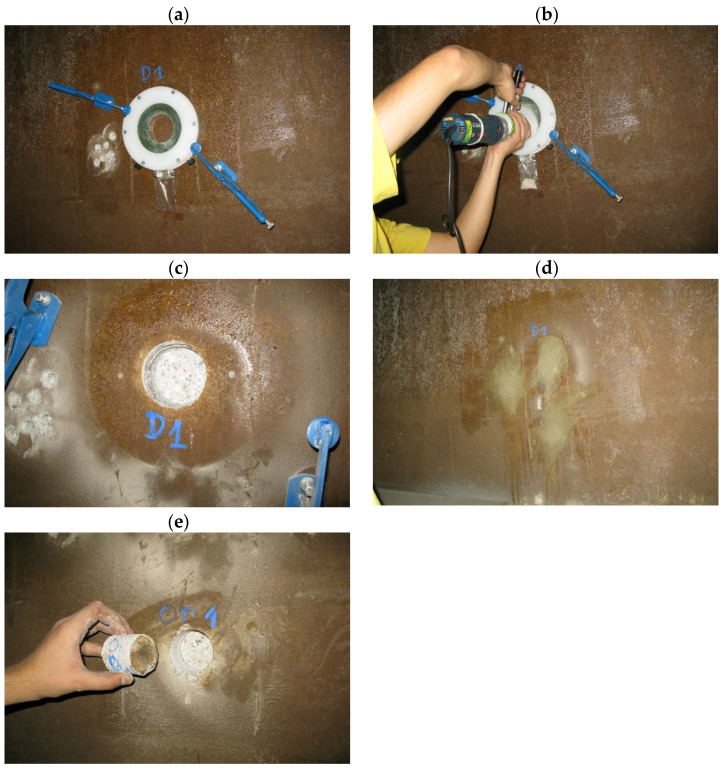
Stages in sample taking by means of the Profile Grinder Kit in the exemplary case of girder VI: (**a**) fixing guide; (**b**) grinding off the concrete layer; (**c**) view of the hole left after taking samples; (**d**) filling holes left after taking samples; (**e**) concrete cores.

Samples of ground concrete from the lagging thickness (five in the zone of highest chloride corrosion risk and four of lower risk) and core borings were taken from nine structural elements of the aluminium foundry hall (Figure 3) located in different parts of the hall. The number of samples taken was related to the accessibility of the sampling locations and the concern for weakening of the structural elements. The authors are aware that the number of samples taken may be statistically questionable. Crushed concrete was taken from seven depths every 6 mm (giving a total of 42 mm) automatically using a specialised Profile Grinder Kit, Houston, TX, USA, (Figure 4a–d) without the use of cooling fluids. Nine core holes were also drilled ‘dry’. The samples were stored in sealed containers to protect them from changes in chemical composition and moisture content.

Samples of ground concrete were taken from the webs of two roof girders (girder VI—samples D_1_ and girder X—samples D_2_), three ribs of the TT roof slabs (between axes 10-11/E-F—samples P_1_ at the vent, in the field between axes 11-12/F-G—samples P_1+_ and in field 9-9/G-H—samples P_2_), two columns (column F11—samples S_1_ and column G09—samples S_2_), and two curtain walls (wall between columns E10 and E11—samples SC_1_ and wall between columns H08 and H09—samples SC_2_). Using a 50 mm diameter coring drill, concrete core borings of approximately 40 mm were drilled from the web of girders VI and X (samples OD_1_ and OD_2_, respectively), from the ribs of three roof slabs (in the field between axes 10-11/E-F—sample OP_1_ and in the field 11-12/F-G—sample OP_1+_ and in the field 8-9/G-H—sample OP_2_), columns F11 and G09 (samples OS_1_ and OS_2_, respectively), and two curtain walls (between columns E10 and E11—sample OSC_1_ and between columns H08 and H09—sample OSC_2_). The paper presents selected test results for prestressed concrete roof elements of the aluminium foundry hall located close to the aluminium cleaning station (web of girder VI—specimens D_1_ and OD_1_ and rib of roof slab type TT—specimens P_1_ and OP_1_) and distant from this station (web of girder X—specimen D_2_ and rib of roof slab type TT—specimen P_2_).

### 6.2. Testing Methodology of Concrete Cores

**Figure 5:**
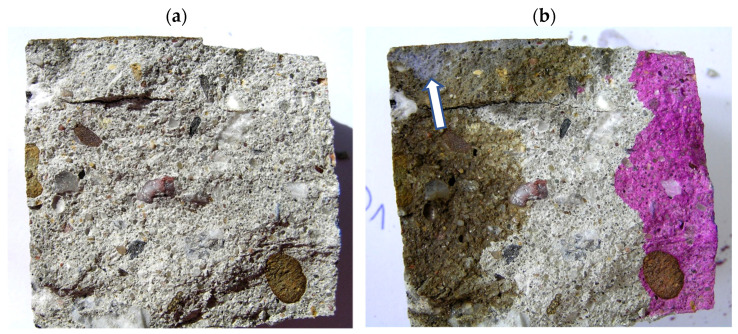
Concrete core (sample OD_1_): (**a**) immediately after splitting and (**b**) after testing with phenolphthalein and AgNO_3_ solution.

**Figure 6:**
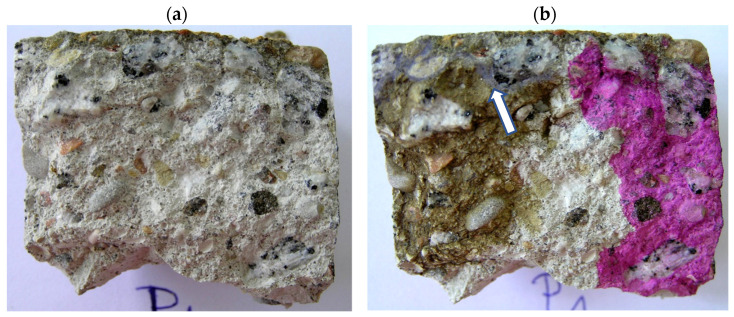
Concrete core (sample OP_1_): (**a**) immediately after splitting, (**b**) after testing with phenolphthalein and AgNO_3_ solution.

The concrete samples in the form of concrete cores (OD_1_ and OP_1_) were split into two halves (Figure 5a and Figure 6a), and the pH and the Cl^−^ ion content and subsequently the composition of the hardened concrete were determined. In order to determine the pH, phenolphthalein solution was poured over the surface of the concrete along the whole height, whereby the sample turned beetroot red along its whole height (Figure 5b and Figure 6b). This result indicates that the pH is higher than 10. AgNO_3_ solution with a concentration of 0.1 mole/cm^3^ was poured over the same half of the sample to preliminarily determine the penetration of Cl^−^ ions into the concrete. In the place where white efflorescence in the form of AgCl deposit appeared (white opalescent efflorescence marked with an arrow in Figure 5b and Figure 6b), free (water-soluble) chlorides in the amount above 0.15% of the cement weight occurred [41]. In at least the same quantities, they penetrate to the depth of 8 mm in girder VI (Figure 5b) and to the depth of 10 mm in the rib of the TT slab (Figure 6b).

An approximate method was used according to instruction [57] to determine the composition of the hardened concrete of the cores on the basis of the apparent density of the concrete, the HCl-insoluble part content in the concrete, and the number of components attached to the binder during its hardening. Because of the irregular shape of the samples, the apparent density ρ_c_ of the concrete, defined as a ratio of the sample’s dry mass to its total volume (together with pores), was determined using the hydrostatic method.

In order to determine the aggregate content in the concrete, it was assumed that the total aggregate content in the concrete was equal to the mass m of the parts insoluble in hydrochloric acid. After the concrete had been comminuted, passed through a sieve with 1 mm mesh, and dried to a constant mass at a temperature of 105 °C, the samples were ground through a sieve with 0.2 mm mesh. Analytical samples, each weighing about 2 g, were subjected to tests using an aqueous solution of hydrochloric acid (1:3) to determine the insoluble part content. The weighed amount was placed in a 250 mL beaker, and 100 mL of the HCl solution was added at room temperature. The beaker’s contents were mixed, and the insoluble residue was ground with a glass rod. After 15 min the liquid from above the deposit was decanted. 50 mL of the HCl solution were poured into the deposit in the beaker, and the whole was placed in a water bath at a temperature of 90 °C for 15 min. Then the beaker’s contents were twice washed with hot water and decanted. 50 mL of 5% Na_2_CO_3_ were poured over the deposit remaining in the beaker the whole was placed in a water bath at a temperature of 90 °C for 15 min, then twice washed with hot water and decanted. 50 mL of water were poured over the remaining deposit, acidified with HCl (1:3) in the presence of methyl orange, additional 3–4 drops of the acid were poured into the neutralised solution, and the beaker’s contents were filtered. The deposit from the filter was washed six times with hot water until the reaction towards chlorides disappeared. The washed deposit was transferred to a weighed porcelain crucible, and after burning the filter, it was roasted at a temperature of 1000 °C. Ultimately, the mass m of the parts insoluble in HCl was determined, and then the insoluble parts percentage content was calculated from the formula: C_ins,%_ = m × 100%. In accordance with the initial assumption, the aggregate content in the concrete was deemed to be directly equal to the analytically determined mass m of the concrete’s parts insoluble in HCl, i.e., C_agg,%_ = C_ins,%_. The results of the analysis are presented as C_agg_ = (C_agg,%_/100) × ρ_c_ in Table 4.

The content (C_att,%_) of the compounds (H_2_O and CO_2_) attached to cement during concrete setting and hardening was determined on the basis of the loss on ignition. The samples were prepared in accordance with the procedure for determining the aggregate content of concrete. In addition, they were ground on a sieve with 0.06 mm mesh. The loss on ignition was determined in an oxidising atmosphere during the roasting of the sample under air at a temperature of 950 °C until a constant sample mass was obtained. The constant mass was determined through successive 15 min long roasting, each followed by cooling and weighing. The percentage loss on ignition was calculated from the formula: C_att,%_ = (m_1_ − m_2_) × 100/m_1_, where m_1_ is the mass of the tested sample and m_2_ is the mass of the roasted sample. The value of C_att,%_ was converted into kg/m^3^ using the formula: C_att_ = (C_att,%_/100) × ρ_c_. Cement content C_cem_ was calculated from the condition: C_cem_ = ρ_c_ − C_agg_ − C_att_ (Table 4).

### 6.3. Testing Methodology of Comminuted Cover Concrete

The samples of powdered concrete taken from different depths of the reinforcement cover differ in their aggregate and cement content. Therefore, the percentage cement content in the concrete was individually determined for each of the powdered samples. For this purpose, for all the concrete samples, the following were determined: moisture content, loss on ignition, and acid-insoluble part content. The measurement results are presented in Table 5 and Table 6.

Water extracts with a powdered concrete/water mass ratio of 1:10 were prepared for measurements of the concrete’s pH. The water extracts had been filtered prior to pH measurements. The pH was measured using an ULAB 2002l pH-meter with an ERH-111 combined electrode. The pH-meter was calibrated within the pH range of 10–13.

After the pH had been measured, a filtrate obtained by washing the deposits with hot distilled water was added to wash out all the water-soluble chlorides. In the filtrates obtained in this way, the chloride ion content was determined using titration with silver nitrite against potassium chromate as the indicator. Before titration, the water extracts had been reduced to a pH of 7–8. At low chloride ion concentrations the determination was carried out using the standard NaCl solution with a concentration of 5 × 10^−4^ mol/dm^3^. The Cl^−^ chloride percentage contents in the concrete were converted to Cl^−^ chloride percentage contents in the cement, taking into account the various cement contents in the particular layers, given in Table 5 and Table 6. The results of the measurements and the conversions are presented in Table 7 and Table 8.

Powdered concrete samples for testing the HNO_3_-soluble chloride ion content were prepared in accordance with standard [55]. After the pH had been reduced to 4, measurements were performed using an Orion 94–17BN ion-selective electrode and a Thermo Orion 4STAR meter. The acid-soluble Cl^−^ chloride contents in the concrete were converted to Cl^−^ chloride percentage content relative to the mass of the cement contained in the concrete of a particular layer. The results are presented in Table 7 and Table 8.

The experimentally determined chloride contents in the cover concrete of the investigated pre-tensioned concrete roof girders and TT slabs (Table 7 and Table 8) indicate that the Cl^−^ ions content in the superficial layer of the concrete of the elements located near the station for purifying liquid aluminium is much higher than in the superficial layer of the elements located as far away as possible from this station. These differences dwindle at depths greater than 15 mm.

## 7. Analysis and Discussion of Reinforcement Cover Concrete Test Results

The tests of the concrete taken from the reinforcement cover indicated a pH of 12.5 in the layers situated deeper than 6 mm from the surface of the structural members (Table 5 and Table 6). Owing to its high pH, the concrete protects the steel in the reinforced concrete and prestressed concrete members against oxygen corrosion caused by the atmosphere prevailing in the shed. The moisture content of the concrete cores (Table 4) taken from the same structural members (girder VI and the rib of the TT slab over the station for purifying liquid aluminium) as the powdered concrete samples is at the level of the moisture content of concrete serving under air, whose relative humidity amounts to about 60%. Thus, the concrete is not damp, and in the inner layers of the girders and the TT slabs, it is even dry (Table 5 and Table 6). The concrete’s relatively high losses on ignition (Table 5 and Table 6) are due to the high cement content in the concrete (C_cem_ = 445 ÷ 447 kg/m^3^—Table 4).

The considerably higher moisture content of the concrete in the 0–6 mm thick outer layer of the cover in the roof girders (Table 5) and in the ribs of the TT slabs (Table 6) can be due to the buildup of moisture on the surface of the concrete as a result of a repair stoppage (the furnaces are shut down). The drop in the temperature of the structure and the rise in the relative humidity of the air in the shed resulting from the inflow of outdoor air caused water vapour condensation on the structural members and the penetration of water with dissolved chloride salts coming from the dusts sticking to the structure’s surfaces (the dusts contain about 20% of Cl^−^ ions—Table 3) into the concrete.

The water-soluble or HNO_3_-soluble Cl^−^ ion contents (Table 7 and Table 8) measured for the particular layers of the concrete cover indicate that most of the chlorides contained in the superficial layer of the concrete are soluble in water. The chlorides come from the atmosphere of the foundry shed, mainly from the process dust after the structure becomes damp. The determined water-soluble and acid-soluble chloride contents in the concrete cover are similar (Table 7 and Table 8).

## 8. Determination of the Theoretical Service Life of Structural Members

For a structure already built and in service in a chlorine environment, it is possible to determine the theoretical service life (i.e., the time after which the concentration of chloride ions near the reinforcement will reach the critical value C_lim_) on the basis of the distribution of chloride content in the reinforcement concrete cover. For this purpose, Fick’s law I and II are used. Fick’s law I defines the relation between diffusing substance flux J(x) (i.e., the amount of substance flowing in a time unit through a unit surface perpendicular to this flux) and the substance concentration gradient:

(1) $$J\left(x\right)=-D_{a}\frac{𝜕C(x,t)}{𝜕x},$$

where:

J(x)—the flux of chlorides moving unidirectionally in a half space,
$D_{a}$—the coefficient of chloride diffusion in concrete,
t—the time of chloride penetration into concrete,
x—the distance from the surface of the concrete to the layer with chloride concentration C(x,t),
C(x,t)—the chloride concentration at distance *x* from the surface of the concrete after structure in-service time *t*.

Fick’s law II describes the relationship between the local rate of changes in the concentration of a diffusing substance and the gradient of its concentration. Assuming that the transport of chlorides takes place unidirectionally in a half space, according to Fick’s law II, the unsteady flow of the flux of chlorides is defined by the relation:

(2) $$\frac{𝜕C\left(x,t\right)}{𝜕t}=-D_{a}\frac{{𝜕}^{2}C\left(x,t\right)}{𝜕x^{2}}.$$

Equation (2) can be solved when one assumes the following boundary conditions: $C\left(x,t\right)=C_{0}$ for x = 0 and *t* > 0 and $C\left(x,t\right)=0$ for x > 0 and *t* = 0. Then one will get function $C\left(x,t\right)$, being the particular integral of Equation (2), described by the equation:

(3) $$C\left(x,t\right)=C_{0}\left[1-erf\left(z\right)\right]=C_{0}\left[1-erf\left(\frac{x}{2\sqrt{D_{a}t}}\right)\right]$$

where:

$C_{0}$—the concentration of chlorides in the superficial layer of the concrete,
erf⁡(z)—an error function (an error integral), which can be read from appropriate tables [58],
$z=0.5\frac{x}{\sqrt{D_{a}t}}$—the argument of the error function.

Equation (3) is valid assuming that the concrete initially contains no chlorides, the diffusing chloride ion concentration measured on the concrete’s surface is constant and amounts to *C*_0_, and the diffusion coefficient *D_a_* is constant over time and does not change across the concrete layer thickness. In reality, chloride ions can penetrate via pure diffusion only in concrete that is completely and permanently saturated with water. As described earlier, in most situations, other transport mechanisms (e.g., capillary rise) contribute to chloride penetration, while bonding with the cement paste components can change the concentration of free chloride ions in the pore solution. Nevertheless, a few studies showed that even in exposure conditions in which the transport of chlorides occurs as a result of phenomena other than diffusion, the experimentally measured profile can be fitted using Equation (3), provided that appropriate *C*_0_ and *D_a_* values are calculated [12,14]. However, Marchand J. and Samson E. [59] are of different opinion. They think that it is improper to use Fick’s diffusion law to analyse the penetration of chlorides into the structure of concrete since the theoretical Fick model is based on many simplified assumptions that do not reflect the actual behaviour of cementitious materials or the environment to which building structures are exposed. Despite the doubts expressed in the paper [59] and the limitations described above, methods based on the theoretical Fick model are widely used, e.g., [60,61,62,63], precisely because of their simplicity. More sophisticated models require many input parameters, which are sometimes difficult to obtain in the field.

The test results for the chloride content in the cover concrete were plotted depending on the distance from the sample’s surface and approximated with function $C\left(x,t\right)$ from Equation (3) through the proper choice of $C_{0}$ and $D_{a}$ values to best fit the shape of this function to the experimental results. The fitting of function $C\left(x,t\right)$ to the experimentally determined Cl^−^ ion content in the concrete of the 0 ÷ 6 mm superficial layer (the zone of the capillary rise of the chlorides dissolved in water), the 6 ÷ 18 mm intermediate layers (the capillary rise and diffusion zone), and the deepest 18 ÷ 42 mm layers (the zone of the dominant diffusion of Cl^−^ ions) turned out to be impossible because of the unstable diffusion of chlorides in the concrete. According to [64], the diffusion coefficient $D_{a}$ can be considered to be constant only in the case of old concrete, i.e., 10–20 or more years old (the material has stabilised).

**Figure 7:**
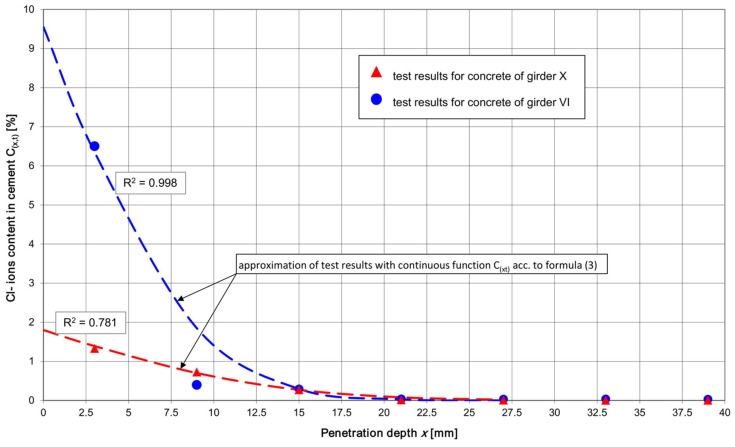
Total content of acid-soluble Cl^−^ ions in cement depending on depth x of chloride penetration into reinforcement cover of girder VI located near station for purifying liquid aluminium and of girder X located as far away as possible from this station.

**Figure 8:**
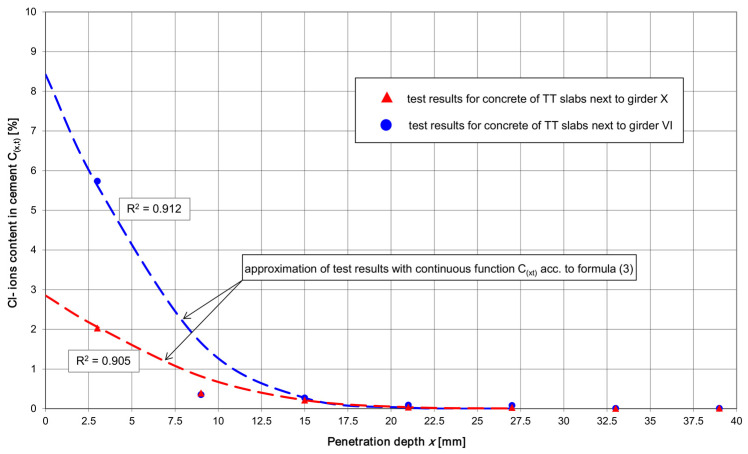
Total content of acid-soluble Cl^−^ ions in cement depending on depth x of chloride penetration into the reinforcement cover of the rib of the TT roof slab located, respectively, near the station for purifying liquid aluminium and as far away as possible from this station.

Function $C\left(x,t\right)$ was separately fitted to the test results for the 0 ÷ 18 mm zone (i.e., the zone of the very strong effect of the periodical capillary rise of the chlorides dissolved in the water and the diffusion of Cl^−^ ions in concrete with a normal moisture content) and for the 19 ÷ 42 mm zone of Cl^−^ ions diffusion in the concrete with a relatively low moisture content. Although these zones overlap at a depth of 12 ÷ 24 mm, this did not hamper the interpretation of the results for the purpose of using function $C\left(x,t\right)$ to calculate the theoretical life spans t_ch_ of the shed’s roof girders and TT slabs. The graph of function $C\left(x,t\right)$ for pre-tensioned roof girders VI and X at time *t* = *t_ex_* (or *t = t_m_*), fitted in the 0 ÷ 18 mm interval to the test results, is shown in Figure 7, while the graph for the TT pre-tensioned concrete slabs is shown in Figure 8.

For samples D_1_ and D_2_ and P_1_ and P_2_, the Cl^−^ ion content in the concrete surface layer $C_{0}$ was determined from the direct fitting of the curve of function C(x,t) to the test results for the chloride content in the concrete. The known value of $C_{0}$ the value of error function erf⁡(z) was calculated from the formula:

(4) $$\mathrm{erf}\left(z\right)=1-\frac{C(x,t)}{C_{0}}$$

For the calculated value of the error function erf⁡(z) the value of argument *z* was read from the table given in [58]. By rearranging Formula (3), the following formula for diffusion coefficient *D_a_* was obtained:

(5) $$D_{a}=\frac{x^{2}}{4\;z^{2}\;t}$$

where *t* is the time for which the structure has so far been in service in the chlorine environment.

Since the launch of partial production until the day the concrete samples were taken, a maximum *t_ex_* = 63 months = 5.25 years had passed. During that period, the concrete structure had been exposed to the gradually increasing concentration of chlorides in the shed’s atmosphere and to their maximum concentration after the full commissioning of all the equipment. Therefore, it was proposed to introduce a coefficient of chloride diffusion into the calculations (Formulas (4) and (5)) for the different periods of the structure’s service life so far (maximum period *t = t_ex_* = 5.25 years and average period *t = t_m_* = 4.0 years). The calculated values of diffusion coefficients *D_a_*_,*ex*_ for *t = t_ex_* = 5.25 years and *D_a_*_,*m*_ for *t = t_m_* = 4.0 years are presented in Table 9.

Knowing the values of the coefficients *D_a_*_,*ex*_ and *D_a_*_,*m*_ of chloride diffusion in the concrete and the function $C\left(x,t\right)$ curves, it was possible to determine the theoretical service life spans (time-until-chemical-breakdown *t_ch_*_,*ex*_ and *t_ch_*_,*m*_) of the particular girders and TT slabs from the formula:

(6) $$t=\frac{x^{2}}{4\;z^{2}\;D_{a}}$$

The determined time-until-chemical-breakdown *t_ch_*_,*ex*_ and *t_ch_*_,*m*_ (Table 10) is understood as the time after which the chlorides concentration in the layer next to the reinforcement will reach the critical value *C_crit_*.

As demonstrated by [21], the chloride threshold *C_crit_* depends on many factors. The main factors identified were the electrochemical potential of the steel, the pH of the pore solution in the concrete, and the presence of voids at the steel-concrete contact. The electrochemical potential of the steel is mainly linked with the concrete’s moisture content, which determines the accessibility of oxygen to the surface of the steel. In structures exposed to the action of the atmosphere, oxygen can easily reach the steel’s surface through pores filled with air. When the concrete is saturated with water, the transport of oxygen to the steel is small. It has been observed that pitting corrosion can take place only above the critical ratio of chloride ions to hydroxyl ions [12]. Therefore, the chloride threshold is a function of the pH of the pore solution, which depends on the kind of cement and the additions. It has also been found that the chloride threshold depends on the presence of macroscopic voids in the concrete in the vicinity of the steel’s surface, which normally occur in real structures due to incomplete consolidation. The research presented in [65] clearly shows that a higher chloride content leads to a higher overall corrosion rate under the same conditions.

In the literature [21,66] one can find various values of critical chloride concentration *C_crit_* in the layer near the reinforcement (from very conservative values of 0.17 ÷ 0.25% through 0.4% to as much as 1.6% relative to the mass of the cement [21]. It is most often recognised that the chloride content relative to the mass of the cement depends on the local regulations and amounts to *C_crit_* = 0.4% for reinforced concrete structures and *C_crit_* = 0.2% for pre-tensioned concrete structures [53].

It is commonly known that there are differences in material specifications, concrete cover thickness, and environmental aggressiveness between individual structures or buildings. These differences have a bearing on service life. It has been found [67,68] that the durability of a structure is more sensitive to reinforcement concrete cover thickness than to diffusion coefficient *D_a_* and more sensitive to surface chloride concentration *C*_0_ than to critical chloride level *C_crit_*.

The service life of the pre-tensioned concrete ribs of the TT slabs and the pre-tensioned concrete roof girders is estimated at about 40 years at the structure’s assumed service life so far, *t = t_ex_* = 5.25 years in the current chloride environment. The shortest service life (about 25 years) was obtained for the panels of the top pre-tensioned concrete TT slabs. Calculations for time *t = t_m_* = 4.0 years indicate a reduction in durability by 31% (Table 10). Calculations performed for two different times *t_ex_* and *t_m_* indicate a significant influence of the length of the structure’s hitherto service life on the structure’s predicted service life in the chloride environment (Table 10). With time, the relative difference between *t_ex_* and *t_m_* will decrease. In the future (e.g., in 5 years time). It will be possible to introduce one hitherto-unknown service life (e.g., *t_m_* = 5.25 + 5.0 = 10.25 years) into the calculations of the predicted service life of the structure.

The calculated service lives (Table 10) of the prestressed concrete roof members situated most far away from the chlorination station relative to the members located next to this station are nearly identical in the current chloride environment. Such results of service life calculations for the main structural members of the shed seem surprising considering the clear differences in the values of *C*_0_ and *D_a_* between the members located near and far away from the liquid aluminium chlorination station (Table 10). The small effect of the location of the shed’s structural members on the calculated service life can be explained by the high diffusion resistance of the relatively dry concrete of the cover’s inner layers (Table 5 and Table 6). Despite the very high chloride content in the cover’s superficial layer in the members located near the chlorination station and the lower chloride content in the distant members, the diffusion of chlorides in the deeper layers of the cover proceeds very similarly (Figure 7 and Figure 8). The chlorides coming from the dust accumulated on the surface of the structure are activated once they become damp and the concrete’s moisture content becomes sufficiently high for the diffusion of chlorides from the superficial layer into the concrete to occur. When the concrete’s relative moisture content falls below 75%, the diffusion of the chlorides rapidly declines [54]. The diffusion of chloride ions can take place only in concrete that is at least partially saturated with water (the pores in the concrete should be filled continuously with the pore solution).

Because of the unstable diffusion of chlorides in the outer layer of the cover due to the periodic damping of the concrete’s surface, it was decided to analyse the theoretical service lives of the shed’s particular structural members, taking into account the diffusion of chlorides in the inner layers of the reinforcement cover. Thus, the high chloride concentrations in the 0 ÷ 6 mm layer (Figure 7 and Figure 8), in which the periodic capillary infiltration of chloride salts dissolved in water occurs quite often, were omitted from the analysis.

In order to fit the function *C*(*x*,*t*) described by Equation (3) to the test results for the chloride content in the layers situated at depths greater than 6 mm (i.e., in the zone in which diffusion plays a dominant role), surface chloride concentrations *C*_0_ in the range of 0.6 ÷ 1.0% were assumed. At depth x = 27 mm, *C*(*x*,*t*) = 0.4 ÷ 0.9% (the values correspond to the concrete test results presented in Table 5 and Table 6). For these assumptions, the values of the diffusion coefficients *D_a_*_,*ex*_, and *D_a_*_,*m*_ were calculated using relations analogous to the ones described above. For the calculated coefficients of the diffusion of chlorides in the concrete and the values of *C*_0_ determined from the graphs of functions *C*(*x*,*t*), the predicted service lives of the shed’s particular structural members were determined.

Among functions *C*(*x*,*t*) satisfying the conditions, one can distinguish such functions that adjust themselves to the chloride concentration test results for a specific structural member of the shed. The individual fitting of function *C*(*x*,*t*) to the concrete test results for each structural member would be advisable in the case of considerable differences in cover concrete test results between the particular members depending on their location relative to the liquid aluminium chlorination station. However, it turns out that the experimentally determined values of the chloride content in the deeper layers of the cover of all the tested members are similar regardless of the location of the members relative to the liquid aluminium chlorination station.

All the test results for the chloride content in the cover of the structural members at depths greater than 6 mm can be described by the function *C*(*x*,*t*) according to relation (3), assuming *C*_0_ = 0.8% and *D_a_* = 27.53 mm^2^/year. This function can be regarded as a concrete test result envelope describing the most disadvantageous pattern of chloride content in the cover of the roof structural members. From this envelope, one can calculate the minimum service life of the prestressed members (the roof girders and TT slabs) with regard to the chloride corrosion of the pre-tensioned tendons, which amounts to 28 years for *t_ex_* = 5.25 years or to 21 years for *t_m_* = 4.0 years. The service life of the members with a 45 mm thick cover (the girders and the TT slabs with shear reinforcement) is estimated at a minimum 50 years, regardless of the assumed time *t_ex_* or *t_m_* (provided that there are no cracks reaching the reinforcement, through which chlorides dissolved in water could directly penetrate). For the panel of the TT slab with a 30 mm reinforcement cover, the service life amounts to 36 years for *t_ex_* = 5.25 years or to 27 years for *t_m_* = 4.0 years. The service life values are close to the calculated ones presented in Table 10.

## 9. Conclusions

Following the elements of discussion given in previous sections, we may draw some major issues to be underlined as the main highlights of the presented study:

- The penetration of chlorides into concrete is a complex process, which in many environments is further complicated by temperature cycles and wet-dry cycles occurring in reinforced concrete structures and prestressed concrete structures. Even though there are many experimental studies and theoretical models relating to the transport of chlorides in concrete, the measurement of chloride penetration into concrete is technically difficult. Moreover, it is difficult to assess the durability of a structure on the basis of its chloride diffusivity, in part due to the heterogeneous character of the concrete matrix, the difficulties in producing concrete in a repeatable way, and the probabilistic by nature character of the transport of chloride ions in concrete.
- The research presented in this paper has confirmed the conjectures about the high aggressiveness of the chlorine environment created in the shed aluminium foundry as a result of periodic changes in thermal-dampness conditions, causing the hydration and activation (during, e.g., breakdowns, unplanned downtimes, or repair stoppages) of the process dusts accumulated on the structure. The hydrated chlorides penetrating into the reinforcement cover become the starting material for the diffusion of Cl^−^ ions towards the reinforcement (provided that the concrete has an appropriate relative moisture content in the deeper layers of the cover). According to the shed’s technical documentation, the structural elements were designed for chloride environment XD1 with moderate air humidity [52,53]. The actual conditions that arose in the shed during the repair stoppage are tantamount to the creation of the cyclically wet-dry environment XD3 [52,53]. This is the transition from a moderately wet environment (XD1) to a cyclically wet environment (XD3), and, as is well known, the wetness of the concrete is responsible here for the transport of chloride ions deep into the concrete. This shows how sometimes it is difficult for a designer to properly predict the class of the environment that will act on the concrete and the reinforcement steel.
- The tests of the chloride content in the concrete of the reinforcement cover of the shed’s structural members showed a very high chloride concentration in the superficial layer (down to the depth of 6 ÷ 12 mm), dependent on the location of the member relative to the liquid aluminium chlorination station (Table 7 and Table 8). In the cover’s deeper layers, i.e., those situated at a depth greater than 15 mm, the chloride content in the concrete only to a small degree depends on the location of a given structural member relative to the chlorination station. This can be indirect evidence of the high diffusion resistance of the concrete in the deeper layers of the reinforcement cover (the concrete is dry).
- In the case of the prestressed elements (the girders and the TT slabs), chloride content, *C_crit_* = 0.2%, was found to be exceeded down to the depth of 18 mm. In the cover layers situated at a depth greater than 33 mm, the chloride content fluctuates around 0.02% of the cement mass in the concrete. This means that the concrete in the deeper layers of the cover contains very small amounts of Cl^−^ chlorides, and the structure is currently not at risk from reinforcement chloride corrosion.
- By taking into account the combined phenomena of the capillary infiltration of aqueous solutions of chlorides in the superficial layers of the concrete, the diffusion of chlorides in the deeper situated layers (Figure 7 and Figure 8), and the “pure” diffusion in the internal layers, and omitting the high values of *C*_0_ in the superficial layer, similar values of service life t of the shed’s particular structural members were obtained. Considering the service life so far in the chloride environment: *t = t_ex_* = 5.25 years, the webs of the shed’s girders (taking into account only the conventional reinforcement) have the longest service life, estimated (assuming shrinkage cracks to be absent) to be at least 50 years. For the prestressed elements (the lower flanges of the roof girders and the ribs of the TT slabs), a service life of about 30 years was obtained. In the case of the TT slab’s upper panel (having the thinnest reinforcement cover of all the reinforced concrete elements, amounting to 30 mm), the shortest service life, estimated at about 25 years, was obtained.
- When calculating the above service lives, a constant value of the diffusion coefficient *D_a_* and the longest possible service life so far, *t = t_ex_* were assumed. For mean *t = t_m_*, the total service lives of the structure decrease by about 31% (a too short lifetime of the shed during which commissioning at a variable chloride concentration in the atmosphere took place, has elapsed). It will be possible to state that the above service lives precisely after the shed has been longer in service.
- The service lives of about 30 years of the shed’s pre-tensioned roof elements, estimated for hitherto service life *t = t_ex_* = 5.25 years in the chloride environment, do not correspond to the design assumptions for class S4 structures (the approximate design service life of 50 years [52]). However, because of the simplifying assumptions (making it possible to apply, i.a., Fick’s law) adopted when calculating the service life of the pre-tensioned roof members, one should treat with caution the estimated service lives. After the shed has been in service for a longer time, a similar range of tests (additionally including tests of the panel of the TT slabs characterised by the shortest estimated service life) should be carried out on the reinforcement cover concrete. Thanks to these tests, the intensity of chloride penetration into the reinforced concrete cover over a longer service life than the current service life will be determined. The results of the tests carried out after the shed has been in service for a longer time will make it possible to precisely state the predicted service life of the structure and to take a proper decision about a possible way of protecting the structural members that are at risk from reinforcement chloride corrosion.

The research was conducted in real life conditions, and its merit was somehow determined by the obtained results. Anyway, we believe that the presented information constitutes a valuable supplement to the current “state of the Art” in the discipline and provides valuable practical outcomes.

## Figures and Tables

**Table 1:** Sampling sites.

Sample No.	Site Description	Substrate	Sample Description
1	Girder III bottom flange haunch over station for purifying liquid aluminium	concrete	The sample consists of fine-grained hygroscopic beige powder, crystalline grains lighter in colour, and very fine, darker dust visible under microscope
2	Girder V bottom flange haunch near station for purifying liquid aluminium	concrete	The sample consists of grey powder with fibres present; grains vary in colour from dark grey to beige under a microscope, with visible larger beige laminar inclusions and transparent fibres

**Table 2:** Loss on drying and loss on ignition, water and acid solubility, and pH of water extracts of tested samples.

Sample No.	Loss on Drying, 105 °C, %	Loss on Ignition, 1000 °C, %	Solubility, %		pH of Water Extracts
			Distilled Water	Hydrochloric Acid HCl:H_2_O = 1:2	
1	23.01	29.59	64.3	80.5	5.10
2	7.56	24.61	37.2	62.0	5.38

**Table 3:** Solubility and percentage of selected ions passing into water solutions.

Sample No.	Water Solubility, %		% of Ions Passing into the Water Solution					
	Insoluble	Soluble	Cl^−^	SO_4_^2−^	Ca^2+^	Mg^2+^	Fe^3+^	Al^3+^
1	35.7	64.3	19.66	2.64	11.09	0.31	abs.	abs.
2	62.8	37.2	11.50	4.31	4.44	1.41	abs.	abs.

**Table 4:** Composition of hardened concrete from cores OD_1_ and OP_1_—test results.

Properties	Test Results			
	OD_1_		OP_1_	
	kg/m^3^	%	kg/m^3^	%
Concrete apparent density ρ_c_	2170	100.0	2197	100.0
Aggregate content in concrete C_agg_ and C_agg.%_	1604	73.9	1543	70.2
Content of substances attached during setting and hardening in concrete (loss on ignition) C_att_ and C_att.%_	119	5.5	209	9.5
Cement content in concrete C_cem_	447	20.6	445	20.3
Moisture content of concrete	28.2	1.30	13.4	0.61

**Table 5:** Measurements of concrete moisture content, loss on ignition, cement content, and pH in samples of powdered concrete taken from different depths of reinforcement cover of girder VI (samples D_1_) located over a station for purifying liquid aluminium and of girder X (samples D_2_) located as far away as possible from this station.

Sample Designation		Layer Location, mm	Distance of Layer Middle from Surface, mm	Moisture Content of Concrete, %	Loss on Ignition at 1000 °C, %	pH of Concrete	Cement Content, %
Girder VI	D_1/1_	0 ÷ 6	3	2.26	9.08	11.67	24.36
	D_1/2_	6 ÷ 12	9	0.65	4.51	12.27	19.30
	D_1/3_	12 ÷ 18	15	0.56	3.47	12.48	19.90
	D_1/4_	18 ÷ 24	21	0.54	3.63	12.57	22.74
	D_1/5_	24 ÷ 30	27	0.86	4.13	12.59	22.64
	D_1/6_	30 ÷ 36	33	0.85	4.31	12.59	20.28
	D_1/7_	36 ÷ 42	39	0.95	4.66	12.60	21.89
Girder X	D_2/1_	0 ÷ 6	3	1.69	12.39	12.27	30.86
	D_2/2_	6 ÷ 12	9	0.77	6.82	12.57	22.02
	D_2/3_	12 ÷ 18	15	0.82	6.12	12.58	21.11
	D_2/4_	18 ÷ 24	21	0.83	7.31	12.60	24.64
	D_2/5_	24 ÷ 30	27	0.89	7.64	12.61	21.78
	D_2/6_	30 ÷ 36	33	0.86	7.21	12.60	23.30
	D_2/7_	36 ÷ 42	39	0.86	6.98	12.60	25.93

**Table 6:** Measurements of concrete moisture content, loss on ignition, cement content, and pH in samples of powdered concrete taken from different depths of reinforcement cover of the TT roof slab located over the station for purifying liquid aluminium (samples P_1_) and of the TT roof slab located as far away as possible from this station (samples P_2_).

Sample Designation		Layer Location, mm	Distance of Layer Middle from Surface, mm	Moisture Content of Concrete, %	Loss on Ignition at 1000 °C, %	pH of Concrete	Cement Content,%
TT slab over chlorination station	P_1/1_	0 ÷ 6	3	1.94	14.88	11.68	28.39
	P_1/2_	6 ÷ 12	9	0.75	11.99	12.56	25.51
	P_1/3_	12 ÷ 18	15	0.73	10.52	12.60	26.48
	P_1/4_	18 ÷ 24	21	0.66	8.43	12.60	24.93
	P_1/5_	24 ÷ 30	27	0.59	9.39	12.60	26.27
	P_1/6_	30 ÷ 36	33	0.68	10.86	12.60	30.22
	P_1/7_	36 ÷ 42	39	0.67	8.92	12.60	26.55
TT slab in the opposite corner of shed	P_2/1_	0 ÷ 6	3	1.39	14.8	12.04	27.06
	P_2/2_	6 ÷ 12	9	0.66	12.59	12.56	27.86
	P_2/3_	12 ÷ 18	15	0.75	9.22	12.59	26.00
	P_2/4_	18 ÷ 24	21	0.71	10.09	12.60	28.60
	P_2/5_	24 ÷ 30	27	0.67	9.74	12.60	29.77
	P_2/6_	30 ÷ 36	33	1.42	10.14	12.59	31.64
	P_2/7_	36 ÷ 42	39	0.82	11.27	12.61	30.00

**Table 7:** Measurements of free (water-soluble) Cl^−^ ions content and total (HNO_3_-soluble) Cl^−^ ions content in concrete or binder (cement) in reinforcement cover of girder VI located over station for purifying liquid aluminium (samples D_1_) and of girder X located as far away as possible from this station (samples D_2_).

Sample Designation		Layer Location, mm	Distance of Layer Middle from Surface, mm	Soluble Cl^−^ Ions Percentage Content			
				in Water		in HNO_3_ Acid	
				Cl^−^/Concrete, %	Cl^−^/Cement, %	Cl^−^/Concrete, %	Cl^−^/Cement, %
Girder VI	D_1/1_	0 ÷ 6	3	1.492	6.125	1.584	6.502
	D_1/2_	6 ÷ 12	9	0.232	1.202	0.077	0.399
	D_1/3_	12 ÷ 18	15	0.036	0.181	0.059	0.296
	D_1/4_	18 ÷ 24	21	0.004	0.018	0.0094	0.041
	D_1/5_	24 ÷ 30	27	0.009	0.040	0.0084	0.037
	D_1/6_	30 ÷ 36	33	0.004	0.020	0.0081	0.040
	D_1/7_	36 ÷ 42	39	0.001	0.004	0.0071	0.032
Girder X	D_2/1_	0 ÷ 6	3	0.443	1.436	0.410	1.329
	D_2/2_	6 ÷ 12	9	0.082	0.388	0.160	0.727
	D_2/3_	12 ÷ 18	15	0.001	0.005	0.057	0.270
	D_2/4_	18 ÷ 24	21	0.001	0.004	0.0036	0.015
	D_2/5_	24 ÷ 30	27	0.001	0.005	0.0016	0.007
	D_2/6_	30 ÷ 36	33	0.001	0.004	<0.001	0.004
	D_2/7_	36 ÷ 42	39	0.001	0.004	<0.001	0.004

**Table 8:** Measurements of free (water-soluble) Cl^−^ ions content and total (HNO3-soluble) Cl^−^ ions content in concrete or binder (cement) in reinforcement cover of TT roof slab rib located near station for purifying liquid aluminium (samples P1) and as far away as possible from this stand (samples P2).

Sample Designation		Layer Location, mm	Distance of Layer Middle from Surface, mm	Soluble Cl^−^ Ions Percentage Content			
				n Water		in HNO_3_ Acid	
				Cl^−^/Concrete, %	Cl^−^/Cement, %	Cl^−^/Concrete, %	Cl^−^/Cement, %
TT slab over chlorination station	P_1/1_	0 ÷ 6	3	1.536	5.410	1.628	5.734
	P_1/2_	6 ÷ 12	9	0.107	0.419	0.091	0.357
	P_1/3_	12 ÷ 18	15	0.054	0.204	0.072	0.272
	P_1/4_	18 ÷ 24	21	0.003	0.012	0.023	0.092
	P_1/5_	24 ÷ 30	27	0.003	0.011	0.022	0.084
	P_1/6_	30 ÷ 36	33	0.003	0.010	0.0026	0.009
	P_1/7_	36 ÷ 42	39	0.003	0.011	0.0028	0.011
TT slab in the opposite corner of shed	P_2/1_	0 ÷ 6	3	0.444	1.641	0.550	2.033
	P_2/2_	6 ÷ 12	9	0.023	0.083	0.110	0.395
	P_2/3_	12 ÷ 18	15	0.003	0.012	0.056	0.215
	P_2/4_	18 ÷ 24	21	0.001	0.003	0.011	0.038
	P_2/5_	24 ÷ 30	27	0.001	0.003	0.009	0.030
	P_2/6_	30 ÷ 36	33	0.001	0.003	0.0024	0.008
	P_2/7_	36 ÷ 42	39	0.003	0.010	0.0037	0.012

**Table 9:** Calculated values of the coefficients D_a,ex_, and *D_a_*_,*m*_ of chloride diffusion in concrete in the outer layers of reinforcement cover.

Concrete Sample Designation and Sample Taking Site		*C*_0_	*x*	$C\left(x,t\right)$	*erf*(*z*)	*z* Acc. to [31]	*D_a,ex_*	*D_a,m_*
							mm^2^/year	
		%	mm	%			*t_ex_* = 5.25 Years	*t*_m_ = 4.0 Years
D_1_	Girder VI	9.55	15	0.296	0.9690	1.530	4.577	6.007
D_2_	Girder X	1.80	15	0.270	0.8500	1.018	10.339	13.570
P_1_	TT slab next to girder VI	8.42	15	0.272	0.9677	1.520	4.637	6.087
P_2_	TT slab next to girder X	2.85	15	0.215	0.9246	1.257	6.781	8.900

**Table 10:** Theoretical service life of pre-tensioned concrete roof girders and TT slabs after which chloride concentration in concrete near reinforcement will reach permissible value *C_lim_*.

Concrete Sample Designation and Sample Taking Site			*C*_0_	*C_crit_*	*erf*(*z*) (*C_x_ = C_lim_*)	*z*	*x*	*D_a_*_,*ex*_	*t_ch_*_,*ex*_	*D_a_*_,*m*_	*t_ch_*_,*m*_
			%	%			mm	mm^2^/year	years	mm^2^/year	years
D_1_	Girder VI	prestressing steel	9.55	0.2	0.9791	1.637	45	4.577	41.1	6.007	31.3
		plain-carbon steel		0.4	0.9581	1.439			53.3		40.6
D_2_	Girder X	prestressing steel	1.80	0.2	0.8890	1.122	45	10.339	38.9	13.570	29.6
		plain-carbon steel		0.4	0.7780	0.863			65.7		50.1
P_1_	TT slab next to girder VI	prestressing steel	8.42	0.2	0.9763	1.600	45	4.637	42.6	6.087	32.5
		plain-carbon steel		0.4	0.9525	1.401	30		24.8		19.0
P_2_	TT slab next to girder X	prestressing steel	2.85	0.2	0.9298	1.281	45	6.781	45.6	8.900	34.7
		plain-carbon steel		0.4	0.8596	1.043	30		30.5		23.2

## Data Availability

The original contributions presented in the study are included in the article, further inquiries can be directed to the corresponding author.
